# Supplementary material for: Patient-Centered Communication (PCC) scale: Psychometric analysis and validation of a health survey measure
Source: PLoS One. 2022 Dec 30;17(12):e0279725. doi: 10.1371/journal.pone.0279725 (PMC9803193; doi:10.1371/journal.pone.0279725)
Supplement: Attachment — Submitted filename: Moser response to reviewers.docx [file pone.0279725.s001.docx]

**Patient-Centered Communication (PCC) scale: Psychometric analysis and validation of a health survey measure**

Response to Reviewers

Dear Editor: Thanks for the opportunity to respond to the reviewers’ comments. Below you’ll see the comments and our response to each point in red.

Review Comments to the Author

Please use the space provided to explain your answers to the questions above. You may also include additional comments for the author, including concerns about dual publication, research ethics, or publication ethics. (Please upload your review as an attachment if it exceeds 20,000 characters)

Reviewer #1: This is a really nicely done study that is written clearly. I have only a minor comment. As the measure has such a pronounced ceiling effect (33% of respondents having a "perfect" score, I was hoping the authors could comment on how this compares to ceiling effects of other patient-reported measures of provider communication, such as the CAT (Makoul) or the CAHPS communication items (AHRQ). Have the authors considered what cutpoints might be useful (e.g., perfect scores against all else; three tiers perfect, good, inadequate) to deal with these ceiling effects?

Response: Thank you for this comment. We agree that the PCC showed higher than ideal ceiling effects. However, due to the communication-focused construct, and number of items, this was not unexpected. For example, the PCC ceiling effect is comparable to CAHPS (we’re not familiar with the CAT measure). We have added a reference and additional information about this on pages 15 and 16. Fortunately, the PCC also reported a minimal floor effect (0.2%). Therefore, we can feel comfortable to conclude this validation is sensitive to communication deficits. This has been noted both in the Discussion and Conclusion sections.

We also agree that PCC cut-points and percentiles would be incredibly useful to examine communication-focused research questions. However, our psychometric analyses were designed to evaluate the scale’s item-level properties, and an overall evaluation of the total scale score. We strongly encourage investigators to consider common cut-points and percentiles, based on their proposed research questions and target population and added this information to the conclusion.

Reviewer #2: This high quality manuscript describes the psychometric validation of a 7-item patient-centered communication scale in a large nationally representative sample of U.S. adults. Due to the paucity of well-validated communication scales, this work is a valuable contribution.

Response: We’re very happy to hear this is considered a valuable contribution.

No major concerns. This study appropriately evaluated the psychometric properties of the instrument as could be done with the available data.

Response: We appreciate this positive comment.

Minor concerns: Information about how the CFA fit was to be evaluated (“For the CFA, fit was
determined using the following fit indices: Root Mean Square Error of Approximation (RMSEA,
cut off <.08), Comparative Fit Index (CFI, cut off >.90), Tucker Lewis Index (TLI, cut off >.95),
and Standardized Root Mean Square Residual (SRMR, cut off <.08). We did not report the chi
square statistic fit as research has found that this test is sensitive to large sample sizes”) should be provided in the Methods section instead of Results. Similarly, in the section on discriminant validity, for the point about not reporting p-values for correlation coefficients.

Response: The information noted here was moved from the Results to the Statistical Analysis subsection on pages 9-10 in the Methods section.
